# Non-Aziridination Approaches to 3-Arylaziridine-2-carboxylic Acid Derivatives and 3-Aryl-(aziridin-2-yl)ketones

**DOI:** 10.3390/ijms23115919

**Published:** 2022-05-25

**Authors:** Boriss Strumfs, Kirils Velikijs, Romans Uljanovs, Stanislavs Sinkarevs, Ilze Strumfa

**Affiliations:** 1 Latvian Institute of Organic Synthesis, 21 Aizkraukles Street, LV-1006 Riga, Latvia; boriss@osi.lv or; 2 Department of Pathology, Riga Stradins University, 16 Dzirciema Street, LV-1007 Riga, Latvia; kirils.velikijs@rsu.lv (K.V.); romans.uljanovs@rsu.lv (R.U.); stanislavs.sinkarevs@rsu.lv (S.S.)

**Keywords:** aziridines, 4-isoxazolines, azirines, cyclization, cycloaddition, Baldwin rearrangement

## Abstract

Highly functionalized aziridines, including compounds with aromatic moieties, are attractive substrates both in synthetic and medical areas of chemistry. There is a broad and interesting set of synthetic methods for reaching these compounds. Aziridination represents the most explored tool, but there are several other more specific, less well-known, but highly promising approaches. Therefore, the current review focuses on recently described or updated ways to obtain 3-arylated aziridines via different non-aziridination-based synthetic methods, reported mainly since 2000. The presented methods belong to two main directions of synthesis, namely, cyclization of open-chain substrates and rearrangement of other heterocycles. Cyclization of open-chain substrates includes the classic Gabriel-Cromwell type cyclization of halogenated substrates with amines, base-promoted cyclization of activated aminoalcohols (or its analogues), and the oxidative cyclization of β-dicarbonyls. Rearrangements of other heterocycles are presented as the Baldwin rearrangement of 4-isoxazolines, the cycloaddition of 1.3-dipoles or dienes to 2H-azirines, and the addition of C- and N-nucleophiles to the double bond of azirines.

## 1. Introduction

Finding new potential antiviral, antibacterial, and anti-cancer drugs, as well as the development of efficient methods to synthesize building blocks for them, is one of the most important problems in synthetic and medical chemistry. Due to the high reactivity of the strained aziridine ring, derivatives of aziridine-2-carboxylic acid react with various nucleophilic agents. Therefore they remain interesting synthetic substrates for constructing different amino acids including unnatural amino acids and different heterocyclic compounds [1]. Some derivatives of aziridine-2-carboxylic acid, e.g., imexon, azimexon [2], and leakadine [3], have been explored as anti-cancer immunomodulators.

Highly functionalized, especially with aromatic moieties, aziridine-2-carboxylic acid derivatives are now in the focus of interest because their close analogues—aromatic α,β- unsaturated carboxylic acids, such as caffeic acid [4] and its esters [5]—have demonstrated cytotoxic effects and enhancement of apoptosis in lung carcinoma cells. Their analogue, *p*–coumaric acid, features an anti-angiogenic effect [6]. Angiogenesis is necessary for tumor development. Therefore antiangiogenetic properties also may be a background of antitumor drug design. 

**Scheme 1:**
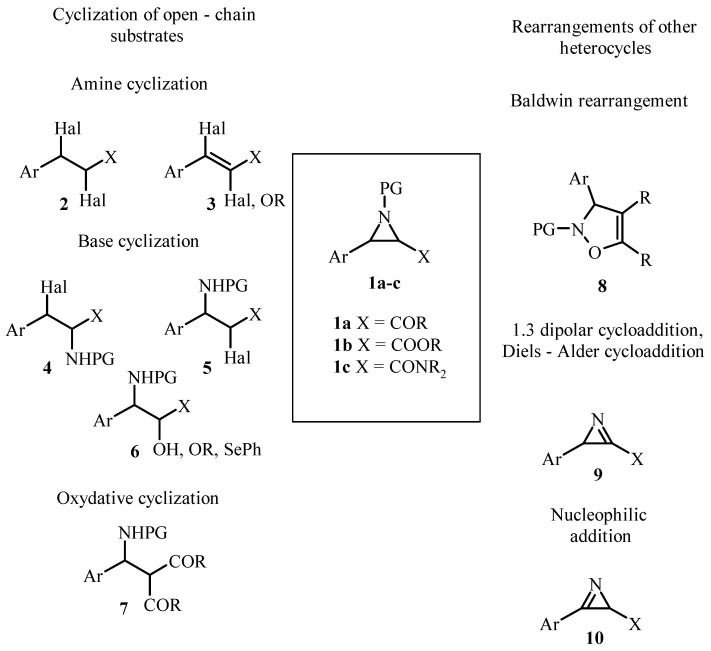
Non-aziridination approaches for aziridines **1a**–**c**.

In this light, the combination of (1) potential properties of specific apoptotic and anti-angiogenic effects of α,β-unsaturated β-arylated compounds and (2) cytotoxic and possible immunomodulating activity of aziridine-2-carboxylates in the same drug-candidate molecule should be a promising direction in the search for new antitumor agents. Based on these considerations, 3-arylated derivatives of aziridine-2-carboxylic acid **1a,b** (Scheme 1) are attractive as potential anticancer and antibacterial drug candidates and building blocks for the development of small-molecule-based, relatively inexpensive medications.

There is a series of general reviews summarizing the recent advances in the synthesis of aziridine-2-carbonyl compounds (Zalubovskis and Ivanova [7]) and general aziridine synthesis and chemistry (Singh [8] and Luisi [9]). The universal methods for the construction of these compounds are via aziridination, elucidated in our previous review [10]. In contrast, this review is focused on selected methods to obtain the same 3-arylated aziridines **1a**–**c** using different, specific approaches. In general, two main directions of synthesis are presented (Scheme 1):

- Cyclization of open-chain substrates;
- Rearrangement of other heterocycles.

Cyclization of open-chain substrates includes the classic Gabriel-Cromwell type cyclization of halogenated substrates **2** and **3** with amines, base-promoted cyclization of activated aminoalcohols or its analogues **4**–**6,** and oxidative cyclization of β-dicarbonyls **7** (Scheme 1).

Rearrangements of other heterocycles are presented as the Baldwin rearrangement of 4-isoxazolines **8**, the cycloaddition of 1.3-dipoles or dienes to 2H-azirines **9**, and the addition of C- and N-nucleophiles to the double bond of azirines **10** (Scheme 1).

In some cases, these methods allow the necessary specific 3-arylated aziridine products to be obtained from easily accessible substrates and may show appropriate chemo- and stereoselectivity.

## 2. Cyclization of Open-Chain Substrates

### 2.1. Classic Gabriel-Cromwell Type Cyclization of Halogenated Substrates

**Scheme 2:**
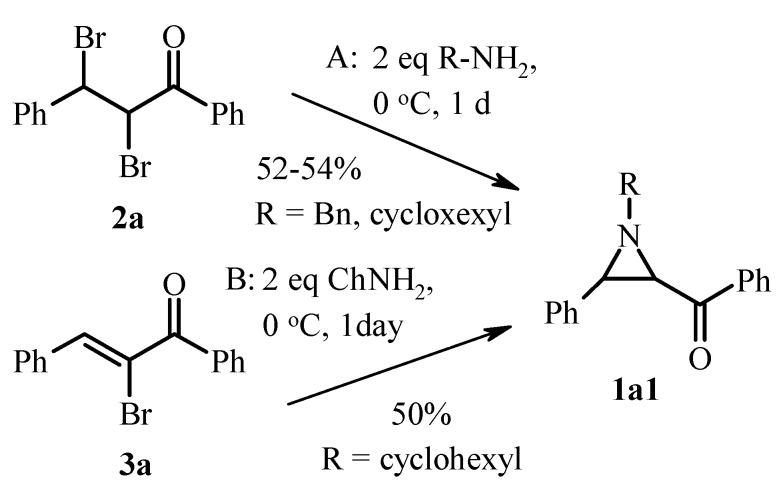
Cromwell cyclization of substrates **2a** and **3a**.

The most popular and well-explored synthetic method for obtaining aziridines in general, especially 3-aryl substituted aziridines including (2-aziridinyl) ketones **1a**, aziridine-2-carboxylates **1b** and carboxamides **1c** is the cyclization of dihalogen derivatives **2** and halogenated olefins **3** with amines. The first 3-aryl-(2-aziridinyl) ketones **1a1** were reported by Cromwell and co-workers in 1943 [11] (Scheme 2).

Aziridine ketones **1a1** were obtained from α,β-dibromo-benzyl-acetophenone **2a** (Scheme 2; path A) or α-bromobenzalacetone **3a** (Scheme 2; path B) in moderate yields by reacting with corresponding amines, in the given example—cyclohexylamine and benzylamine. This is a classical approach, used repeatedly in many studies during XX and XXI centuries. Pathway B is partly like the aziridination reactions in which the double bond in the substrate reacts with an active nitrogen source—nitrene. In the cases discussed therein, the process is different and proceeds through a β-haloamine intermediate. Below in this review, we will focus on the further development and recent more advanced similar methodologies.

**Scheme 3:**
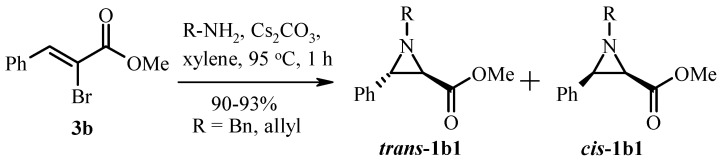
Cromwell cyclization of cinnamates **3b**.

A simple in situ iodination/amination protocol using chalcone type substrates for obtaining type **1a** aziridines was reported in 2001 [12]. The antibacterial activity of these compounds has been discussed. Aziridine ketones **1a** were obtained in 55–57% isolated yields using benzene as a solvent; reactions were performed at room temperature for 1 h. No chromatography was required, as the products were purified by simple recrystallization. Application of similar amination for α-bromo cinnamates **3b** was reported later [13] (Scheme 3). 

**Scheme 4:**
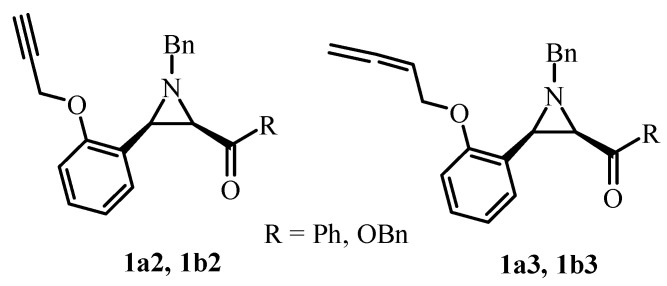
Functionalized aziridine ketones **1a2**, **1a3** and esters **1b2**, **1b3**.

In this case, the yields were excellent, exceeding 90%; aziridines **1b1** were obtained as a mixture of diastereomeres ***trans*-1b1** and ***cis*-1b1,** and the authors noted that it was the first report on the synthesis of cinnamate-derived aziridine esters of type **1b1**. These cinnamate-derived aziridines (**1a**, **1b**) obtained by the given methods were used as templates for the stereospecific synthesis of 2-azetidinones [14] and in the construction of a series of diverse alkaloid molecules [15]. The study [16] demonstrated that the cinnamate, chalcone bromination, or iodination/amine cyclization procedure tolerated another triple bond and allene system into the molecule allowing specific O-propargyl (**1a2**, **1b2**) and buta 2.3-dien-1-yloxy (**1a3**, **1b3**) derived aziridines to be obtained (Scheme 4). 

The simple Cromwell-type aziridine synthesis was successfully used to create aziridine libraries to screen for potential antiplasmodial protease inhibitors [17] and anti-yeast *Candida albicans* agents [18,19].

**Scheme 5:**
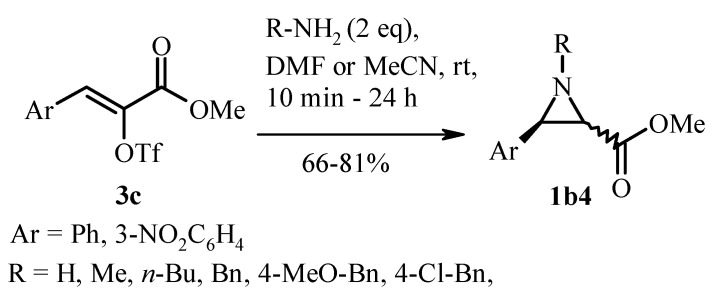
Amine cyclization of vinyl triflates **3c**.

Like halogen, triflate can be used as the leaving group in this type of cyclization. Thus vinyl triflates **3c** [20] (Scheme 5) form aziridines **1b4** in reactions with amines. The best solvents for this process are MeCN and DMF, and the yields of aziridines **1b4** are slightly better in MeCN. The reaction time strongly depends on the nature of vinyl triflate substrate **3c**. Thus, 3-nitrophenyl substituted substrate **3c** requires only 10 min at 0 °C and demonstrates 2.5:1 *trans* selectivity, but in the case of other triflates, the reaction ends only after 24–36 h. 

**Scheme 6:**
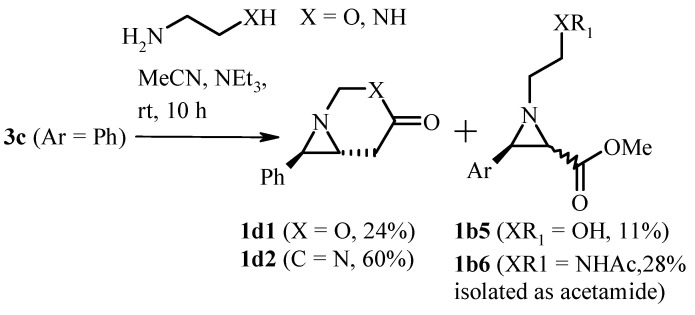
Reaction of vinyl triflate **3c** with ethanolamine and ethylenediamine.

In reactions of phenyl substituted triflate **3c** with ethanolamine or ethylenediamine, interesting bicyclic structures—fused bicyclic lactone **1d1** and lactam **1d2** were obtained in mixtures with the corresponding *cis*-aziridine-2-carboxylates **1b5** and **1b6** [20] (Scheme 6). 

### 2.2. Base-Promoted Cyclization of β-Substituted Amino Substrates

**Scheme 7:**
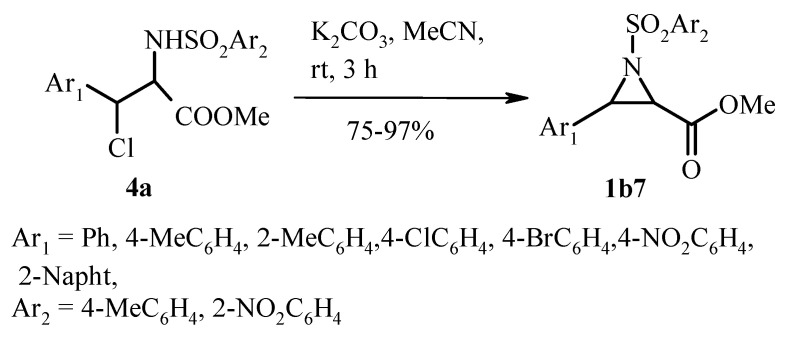
Base cyclization of esters **4a**.

α-Amino-β-halo-esters **4a** derived from corresponding precursors, e.g., cinnamates, undergo cyclization into aziridines **1b7** (without racemization), using TsNCl_2_ mediated aminohalogenation under mild basic conditions in the presence of potassium carbonate [21] (Scheme 7). 

The isolation procedure is also simple and does not require flash chromatography. The efficacy of the protocol has been demonstrated in 11 examples and with seven different β-aryl substituents [21].

**Scheme 8:**
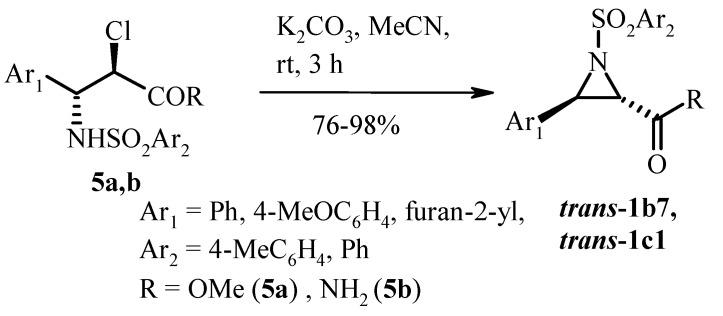
Base cyclization of esters **5a** and amides **5b**.

The base-promoted cyclization into *trans*-3-aryl aziridine-2-carboxylates ***trans*-1b7** and carboxamides ***trans*-1c1** is similar in the α-chloro-β-amino substrates **5a**,**b** [22] (Scheme 8), despite having an opposite structure. Six examples and three different aryl substituents have been reported.

The optimization of this type of aziridine synthesis using a TsNCl_2_-mediated aminohalogenation-cyclization sequence was reported in 2004 for chalcone- and cinnamate-type substrates as “indirect aziridination” [23]. This is a stereoselective one-pot process leading to aziridines **1b** (10 examples); the isolation of aminohalogenation intermediate products **5** was considered unnecessary. 

**Scheme 9:**
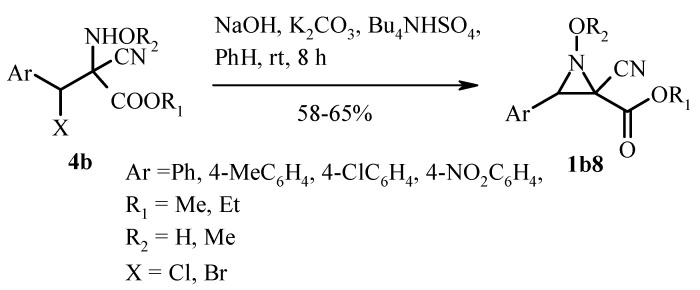
Base cyclization of esters **4b**.

β-Halo-α-aminoesters **4b** derived from corresponding halohydrins were used as the source in the synthesis of specific N-alkoxy-3-arylated aziridine-2-carboxylate **1b8** series [24] (Scheme 9). 

The products **1b8** were demonstrated in 12 examples with good yields. Thus, it appeared to be a reliable and promising method for synthesizing limited access 3-aryl-N-hydroxy and N-methoxy aziridines.

**Scheme 10:**
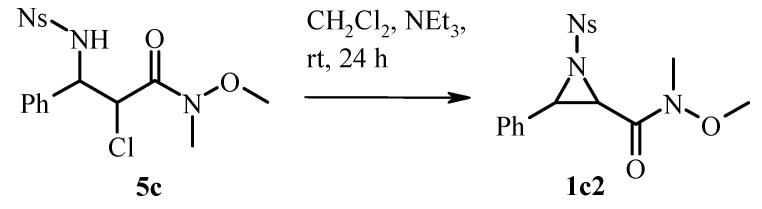
Base cyclization of Weinreb amides **5c**.

3-Arylaziridine Weinreb amides **1c2** can be synthesized in a similar way from the corresponding chlorinated Weinreb amides **5c**. The reaction benefits from the retention of configuration [25] (Scheme 10). 

**Scheme 11:**
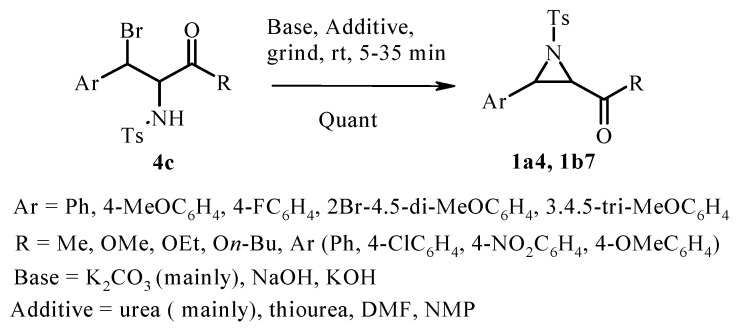
Solvent-free urea-promoted base cyclization of substrates **4c**.

Significant development has been reported simplifying the synthesis of aziridine ketones **1a4** and esters **1b7** from 1.2-vicinal haloamines **4c** [26] (Scheme 11). Base/additive (urea)-promoted cyclization proceeds in solvent-free conditions by grinding components together at room temperature during different time intervals (ranging from 1 min to 1 h), resulting in quantitative chemical yield. 

The said procedure was approved in 18 examples. In 13 of them, products were 3-aryl (2-aziridinyl) ketones **1a4** and 5-3-arylaziridine-2-carboxylates **1b7**.

**Scheme 12:**
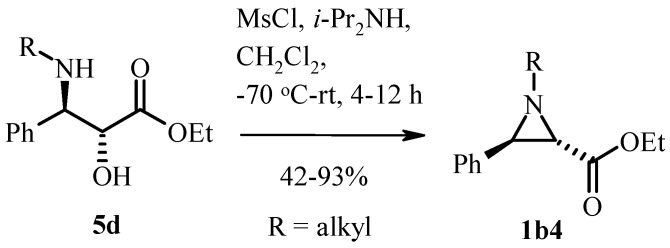
MsCl/base-promoted cyclization of aminoalcohols **5d**.

Aside from halogens, other activators/leaving groups for this type of β-amino compound cyclizations into aziridines were investigated. The methanesulfonyl O-activation of the OH group in aminoalcohol type substrates **5d** allowed a series of N-alkylated *trans*-3-phenylaziridine-2 carboxylates **1b4** [27] to be obtained (Scheme 12). 

The one-pot procedure was illustrated with 12 examples bearing various alkyl type N-substituents. In addition, Boc-protected 3-arylaziridine-2-carboxylates type **1b** were obtained from corresponding amino acids using the same protocol with Ms and Ts O-activation [28].

**Scheme 13:**
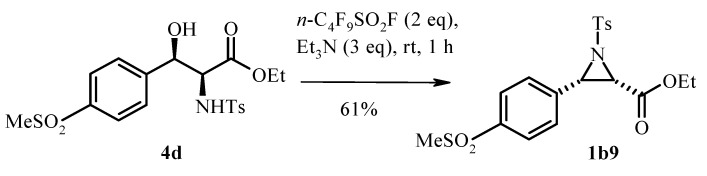
Fluoroalkanosulphonyl fluoride-promoted cyclization of ester **4d**.

The opposite substrate, namely, β-hydroxy-α-aminoester **4d** has been activated with fluoroalkanosulphonyl fluoride for cyclization into 3-aryl aziridine-2-carboxylate **1b9** [29] (Scheme 13). 

**Scheme 14:**
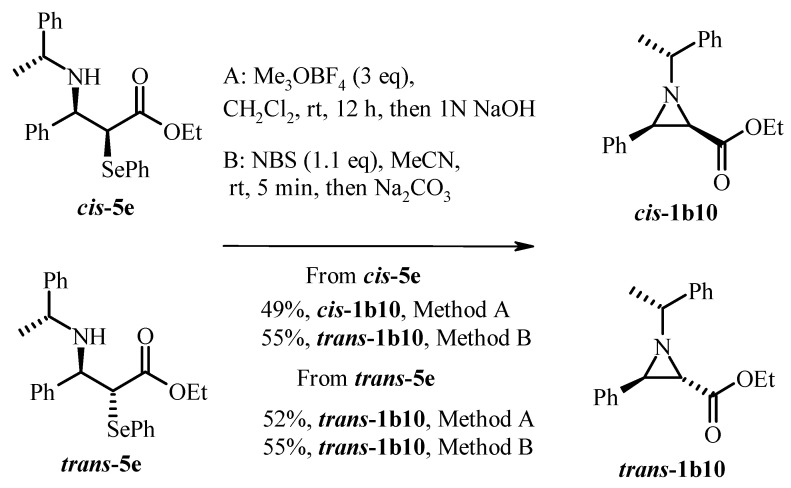
Cyclization of amino selanyl esters **5e**.

In a specific case, the SePh group in amino selanyl esters ***cis*-5e** and ***trans*-5e** appears to be a good activator for selective diastereomeric aziridine-2-carboxylates ***cis*-1b10** and ***trans*-1b10** synthesis [30] (Scheme 14). 

Two different cyclization approaches were examined, using trimethyloxonium tetrafluoroborate (Scheme 14; Method A) and N-bromosuccinimide (Scheme 14; Method B). The configuration of the aziridine product was dependent on the cyclization approach.

### 2.3. Oxidative Cyclization

**Scheme 15:**
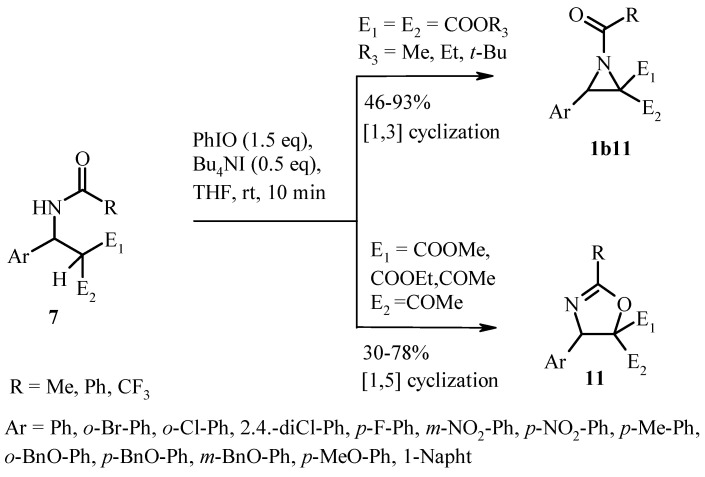
Oxidative cyclization of substrates **7**.

Aminoalkylation adducts of the activated methylene compound **7** undergo [1,3]-oxidative cyclization into N-benzoyl aziridines **1b11** [31] (Scheme 15) in the presence of iodosobenzene and a catalytic amount of tetrabutylammonium iodide. Another possible pathway is represented by the [1,5]-oxidative cyclization leading to oxazolines **11**. The catalytic role of tetrabutylammonium iodide is to depolymerize and thus activate the polymeric iodosobenzene. If malonates are applied as the active methylene compound **7**, the reaction proceeds towards aziridines **1b11** (17 examples). In contrast, if substrate **7** is represented by ketoesters and/or diketones, oxazolines **11** are obtained as the main products. At the same time, the corresponding aziridines **1b11** are observed as minor products in the reaction mixture (eight examples). 

In the reported conditions, the mutual conversion of aziridines **1b11** and oxazolines **11** does not occur. Otherwise, as discussed below, 4-isoxazolines may work as aziridine precursors in the Baldwin rearrangement.

Summarizing this overview on classical and well-explored cyclization methods, some general features are evident, also setting the directions for future development of these approaches to increase the sustainability and simplicity of synthetic procedures:

- Excluding chromatography in the isolation step [13,21];
- Solvent-free reaction conditions [26];
- One-pot activation-cyclization sequences [28];
- Room temperature for reaction in most of examples.

## 3. Aziridines from Other Heterocycles

### 3.1. Aziridines from Isoxazolines. Baldwin Rearrangement

**Scheme 16:**
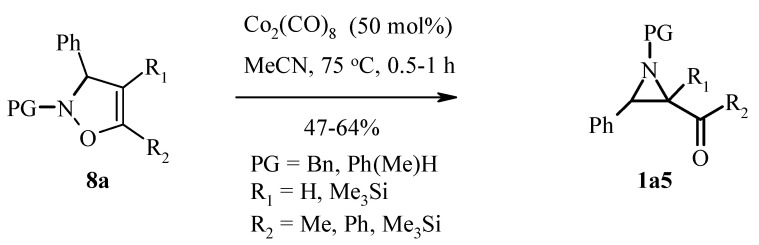
Dicobalt octacarbonyl-promoted Baldwin rearrangement of 4-isoxazolines **8a**.

An interesting method for obtaining target aziridines **1** is the transformation of 5-membered ring substrates, especially 4-isoxazolines **8**. The essence of this method is the cleavage of the N-O bond in these substrates, followed by a subsequent valence rearrangement. At first, this approach to 2-acylaziridine synthesis was demonstrated by the Baldwin team in 1968 [32]. One of the earliest attempts to convert this rearrangement into a practical method was demonstrated in 2002 by Ishikawa group [33]. Otherwise-stable isoxazolines **8a** in the presence of dicobalt octacarbonyl turn into highly functionalized (including aryl substituents) aziridines **1a5** in moderate to high yields (47–64%) (Scheme 16). Ten examples have been described. 

Using optically pure substrate **8a** with α-phenylethyl N-protection instead of benzyl- allows for obtaining a single isomer of aziridine **1a5**. Therefore, a possibility of a general strategy of chiral 3-aryl aziridine **1a5** synthesis from 4-isoxazolidinones **8a** has been demonstrated.

**Scheme 17:**
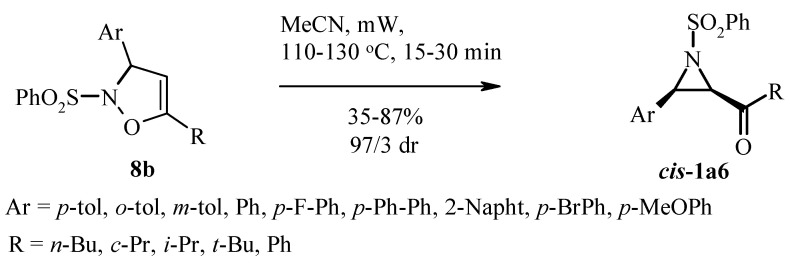
Microwave-mediated Baldwin rearrangement of 4-isoxazolines **8b**.

Using electron acceptor N substituent (N-benzenesulfonamide) on 4-isoxazoline substrate **8b** (Scheme 17) in combination with microwave treatment without catalyst was reported as a useful method for the synthesis of 3-arylated aziridines ***cis*-1a6** [34]. Remarkably high *cis*-selectivity (~97%) was shown. 

The evaluation of different solvents, temperature regimens, and reaction times showed that the best *cis*-selectivity in products ***cis*-1a6**, together with appropriate yields, was reached using acetonitrile as a solvent at 110–130 °C during an approx. 30 min reaction time. Reaction tolerated a wide range of substrates with different aryl substituents (13 examples).

**Scheme 18:**
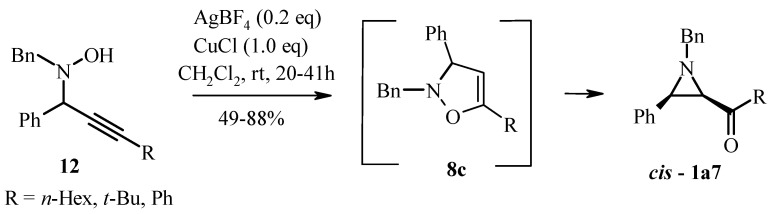
Cascade cyclization-Baldwin rearrangement reaction of hydroxylamines **12**.

4-Isoxazolines **8c** as intermediates in cyclization-Baldwin rearrangement cascade reaction allows the synthesis of *cis*-2-acylaziridines ***cis*-1a7** from N-(propargylic) hydroxylamines **12** [35] (Scheme 18) in a one-pot procedure at room temperature. 

The cyclization of hydroxylamine **12** into isoxazoline intermediate **8c** was catalyzed by AgBF_4_ and Baldwin rearrangement by copper salt. Screening copper salt additives showed that the best additive is CuCl. Moderate to high yields and *cis*-selectivity in β-phenyl aziridines **1a7** has been reported in four examples.

**Scheme 19:**
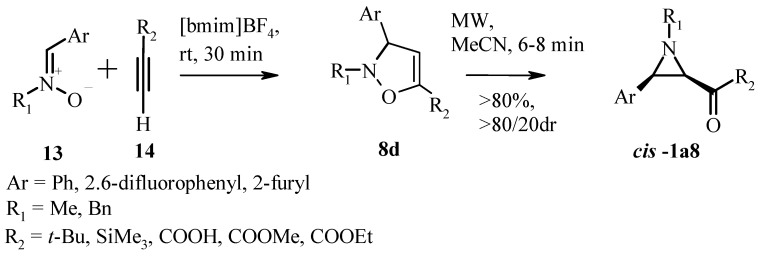
Synthesis and Baldwin rearrangement of 4-isoxazolines **8d**.

Finally, an effective and highly stereoselective synthesis of β-arylated aziridines ***cis*-1a8** and ***cis*-1b12** has been developed, constructing the necessary N-substituted 4-isoxazolines **8d** from nitrones **13** and alkynes **14** through 1,3-dipolar cycloaddition in ionic liquid with subsequent microwave treatment of obtained isoxazolines **8d** in acetonitrile leading to Baldwin rearrangement into aziridines ***cis*-1a8** or ***cis*-1b12** [36] (Scheme 19). The initial nitrones **13** were obtained in the reaction of corresponding aldehydes with hydroxylamines. 

**Scheme 20:**
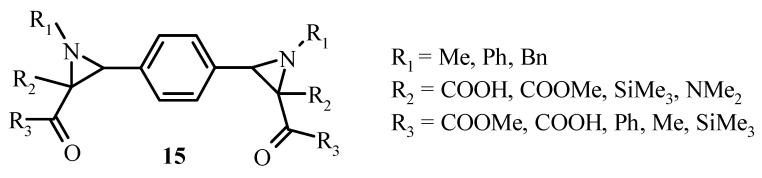
Bis-Aziridines **15**.

Aziridines ***cis*-1a8** and ***cis*-1b12** were obtained in high *cis*-selectivity, as almost single isomers in nine examples. A remarkable feature of this synthesis is the absence of promoting additives and catalysts. Antibacterial properties of obtained aziridines ***cis*-1a8** and ***cis*-1b12** have been discussed. Highly functionalized bis (aziridine) products **15** (eight examples, >70% yields) have been synthesized using the same protocol [37] (Scheme 20). 

In summary, in the case of available isoxazolines Baldwin rearrangement appears as a fast, clean, and easy pathway to reach highly functionalized β-arylated aziridines **1a**–**c** in appropriate chemical yields and high diastereoselectivity.

### 3.2. Aziridines from Azirines

2H-Azirines are considered reactive species and have been employed as substrates in various transformations, including formation of aziridines. Therefore, reactions of azirines seem to be an interesting and useful set of synthetic methods, as discussed below.

#### 3.2.1. 2H-Azirine as 1.3-Dipolarophile

**Scheme 21:**
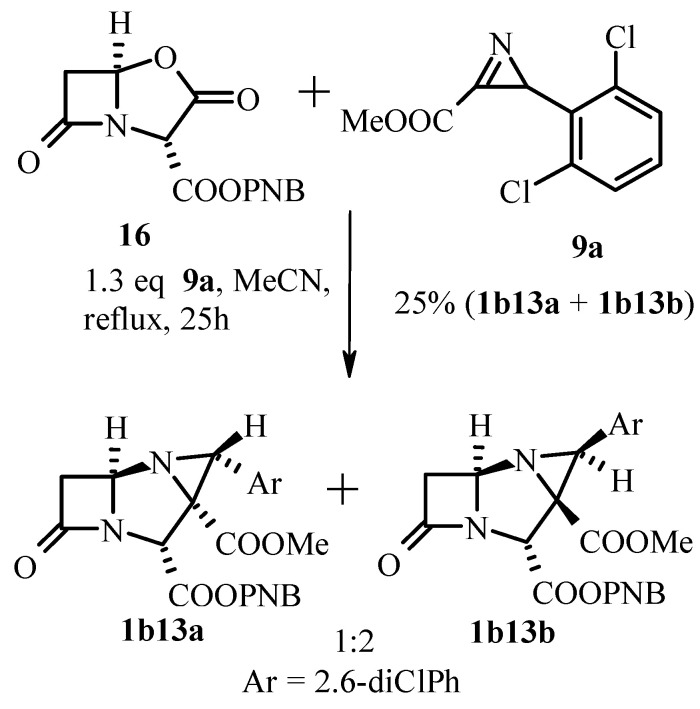
1.3-Dipolar cycloaddition of azirine **9a** to β-lactam **16**.

2H-Azirine **9a** was reported as an efficient substrate for constructing complex aziridine-containing structures **1b13** in reaction with β-lactam adduct **16** [38] (Scheme 21)—a useful precursor for 1-azacepham type structures. Azirine **9a** has been employed as 1.3-dipolarophile towards structure **16** based azometine ylides. Isomers of the obtained adduct **1b13**, namely, the aziridine esters **1b13a** and **1b13b**, are separable by preparative chromatography and can be used in further transformations separately. 

C(2) unsubstituted azirines—analogues of **9a** also are suitable for this reaction and can be generated in situ from the corresponding azides.

#### 3.2.2. Azirines as Dienophiles

**Scheme 22:**
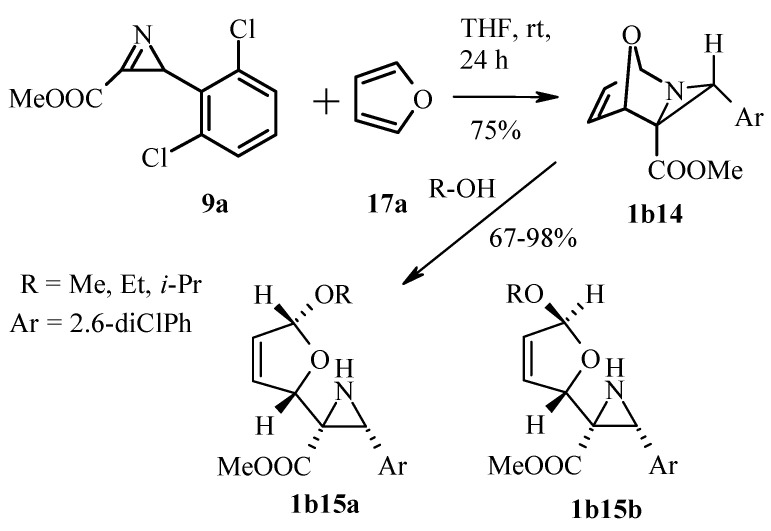
Diels-Alder cycloaddition of azirine **9a** to furan **17a**.

**Scheme 23:**
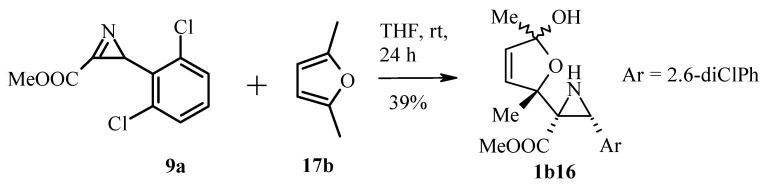
Cycloaddition reaction of azirine **9a** and 2.5-dimethylfuran **17b**.

**Scheme 24:**
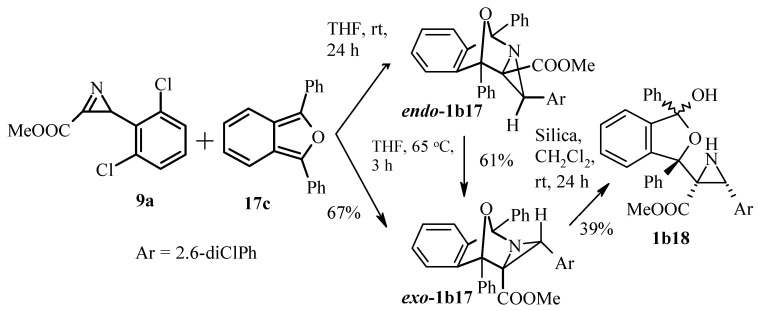
Cycloaddition reaction of azirine **9a** and 1.5-diphenylbenzofuran **17c**.

A series of studies by the Alves group established that 2H-azirines **9**, especially aryl substituted azirine **9a** served as potent dienophiles in Diels-Alder reactions, forming highly functionalized tri- and tetracyclic aziridines bearing β-aryl substituents. Thus, 2H azirine **9a** reacts with furan ring-based dienes: furan **17a** [39], 2.5-dimethylfuran **17b** and 1.3-diphenylbenzofuran **17c** [40] to form highly functionalized 3-arylated aziridine-2-carboxylates **1b14**–**1b18** (Scheme 22, Scheme 23 and Scheme 24). Reaction with furan **d4** leads to aziridine adduct ester **1b14** [39,40] (Scheme 22). Nucleophilic ring opening of aziridine **1b14** with alcohols forms dihydrofurane-substituted aziridine isomers **1b15a** and **1b15b** (three examples). Consequently, the oxazoline ring in adduct **1b14** is more reactive toward O-nucleophiles than the aziridine ring, and it is a promising pathway to obtain specific 2.2 -disubstituted 3-arylated aziridines of type **1b15**. 

Isomers **1b15a** and **1b15b** are separable by flash chromatography.

In the reaction of azirine **9a** with 2.5-dimethylfuran **17b**, only a hydrolysis product—dihydrofurane-substituted aziridine **1b16**—was isolated in moderate yield after chromatography as a mixture of isomers (Scheme 23). 

In contrast, 1.3-diphenylbenzofuran **17c** in reaction with azirine substrate **9a** forms two separable tetracyclic aziridine adducts ***endo*-1b17** and ***exo*-1b17** [40] (Scheme 24). Observations show that ***endo*-d5c1** is the kinetic product, as it is the first to precipitate from the reaction mixture by crystallization (no chromatography is required for the isolation of this product). Further crystallization yielded a mixture of ***endo*-1b17** and ***exo*-1b17**. *Endo* product can be transformed into thermodynamic *exo* product **1b17** by heating in THF for 3 h. 

Hydrolysis of adduct ***exo*-1b17** yielded a pure sample of 3-aryl 2.2-disubstituted aziridine **1b18**.

**Scheme 25:**
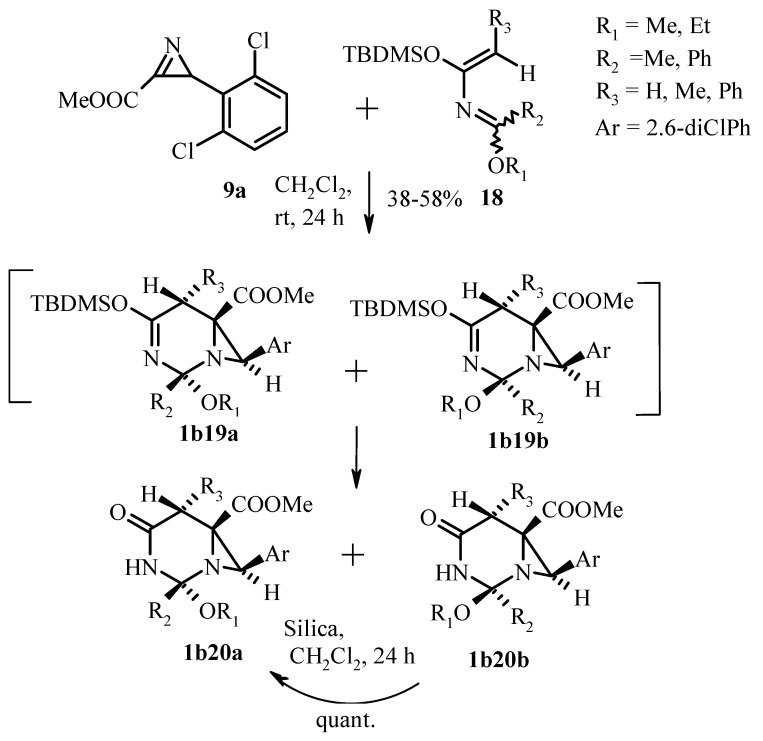
Reaction of 2-azadienes **18** with azirine **9a**.

2-Azadienes **18** react with azirine **9a** in a similar manner, forming bicyclic adducts **1b19** [41] (Scheme 25). Reactions are selective: only *endo*- products were observed. The products were isolated after desilylation as bicycles **1b20**. The isolation procedure was very simple—via filtration, no chromatography was required. In some cases (three examples), a mixture of isomers **1b20a** and **1b20b** were obtained, but if R_1_ = Et, R_2_ = R_3_ = Me, a single isomer **1b20a** was obtained as a precipitate. Chemical yields were moderate. As in the previous case, mixtures can be transformed into a single isomer-thermodynamic product **1b20a** by treatment with silica in dichloromethane (a single example). 

**Scheme 26:**
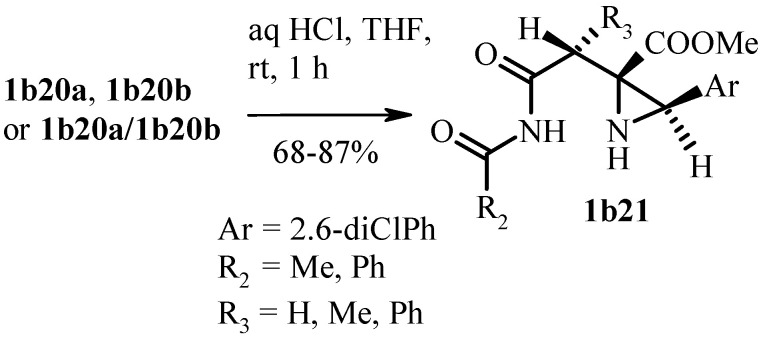
Hydrolysis of aziridines **1b20**.

In the subsequent study [42], the same authors discussed the synthesis of the precursor azadienes **18**, stereochemistry of the aziridine products **1b20**, possible isomerization pathways between compounds **1b20a** and **1b20b,** and their structures in higher detail. The acidic hydrolysis of the aziridines **1b20** forming 3-aryl-NH-aziridines **1b21** (Scheme 26) was reported in five examples. 

Hydrolysis was carried out in acidic media in mild conditions and resulted in aziridines **1b21** with high yields. Both diastereomeres **1b20a** and **1b20b** formed the same products **1b21**. Diastereoselective approaches of these transformations using chiral auxiliaries in aziridine ester moiety were reported in the further study [43].

**Scheme 27:**
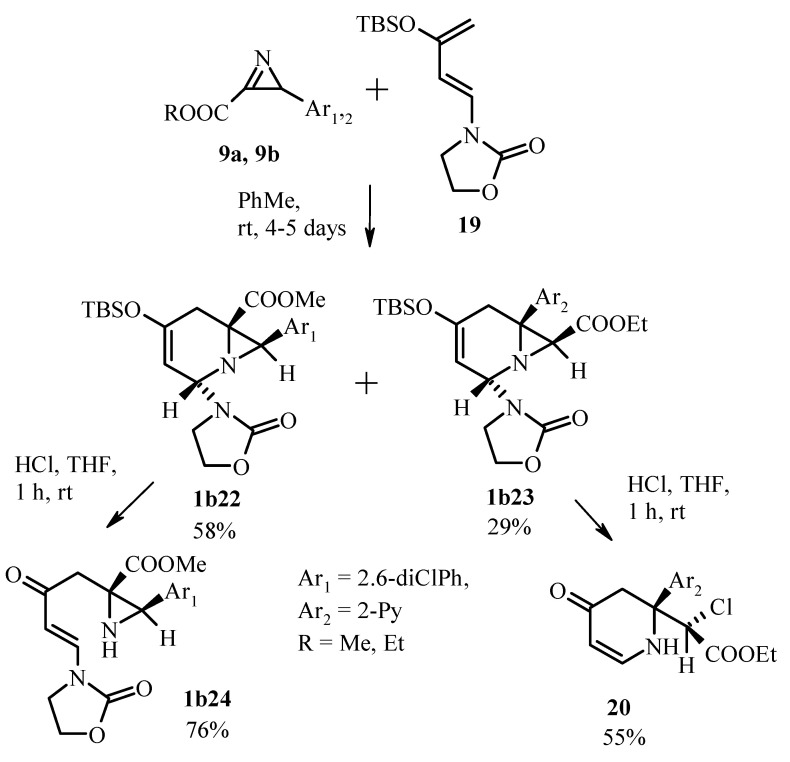
Cycloaddition of diene **19** to azirines **9a** and **9b**.

Exploration of azirine Diels-Alder cycloaddition to dienes was continued with oxazilidin-2-one moiety containing diene substrates **19** [44] (Scheme 27). 

In a reaction with stable 2H-azirines **9a** and **9b**, the cycloadducts **1b22** and **1b23** were obtained. Remarkably, the direction of cycloaddition depends on the aryl substituent. Thus, in case of the 2.6-dichlorophenyl group in the azirine substrate **9a**, a 7-arylated aziridine cycloadduct **1b22** was formed. However, in the case of 2-pyridil substituent (substrate azirine **9b**), a 6-arylated cycloadduct **1b23** was obtained. This shows the possibility of driving cycloaddition via selection of aryl substituents.

As demonstrated in previous studies, products are formed in the *endo*-process. The obtained aziridines undergo acidic hydrolysis in aq HCl-THF media. Different products of hydrolysis were obtained. Thus, 7-arylated aziridine **1b22** yielded aziridine ester **1b24**, while 6-arylated aziridine **1b23** resulted in the product **20** via elimination/ nucleophilic aziridine ring cleavage. 

**Scheme 28:**
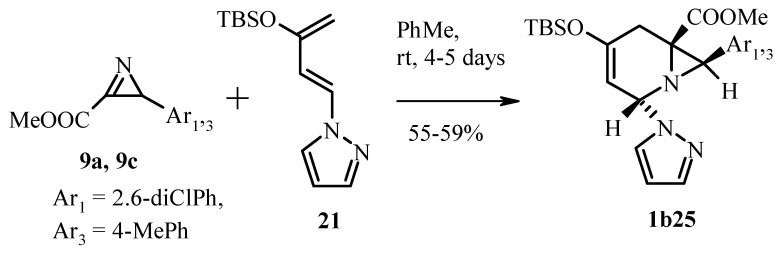
Cycloaddition of diene **21** to azirines **9a** and **9c**.

In the next study [45], the exploration of the synthesis and reactivity of type **1b25** arylated aziridine cycloadducts was continued. Besides 2.6-dichlorophenyl (substrate **9a**) and 2-pyridil (**9b**) substituents in azirine substrate, type **9** *p*-tolyl substituent (substrate **9c**) has been investigated, and a new type of moiety—1-pyrazolyl in the diene **21** was used (Scheme 28). 

Further reactions of **1b25** type cycloadducts have been studied. 

These transformations show relatively easy and direct access to individual isomers of highly functionalized polycyclic β-arylated aziridine products that are difficult to otherwise reach. Remarkably, the reaction conditions and procedures for isolation of products are simple in almost all cases, e.g., cycloaddition reactions were carried out at room temperature.

#### 3.2.3. Nucleophilic Addition to the Azirine Double Bond

**Scheme 29:**
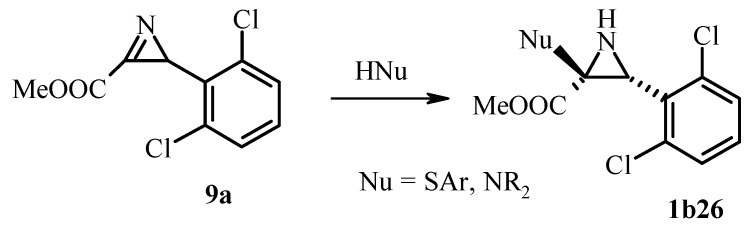
Addition of N- and S-nucleophiles to azirine **9a**.

An significant but not widely explored approach which allows various β-aryl- and heteroaryl-substituted aziridines to be obtained is a protocol based on the addition of nucleophiles (including nitrogen heterocycles) to the 2H-azirine double bond. The initial research by Alves and coworkers demonstrated that 2H-azirine-3-carboxylic ester **9a** formed 3-aryl-2-substituted aziridines **1b26** [46] (Scheme 29). 

**Scheme 30:**
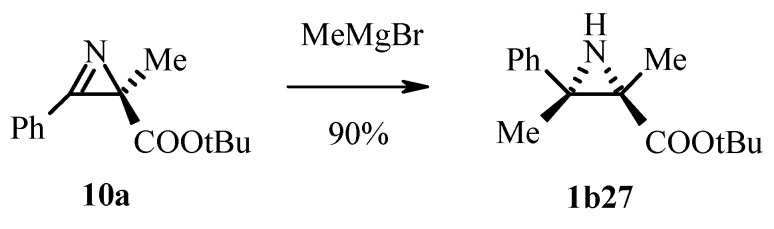
Addition of Grignard reagent to azirine **10a**.

A further study showed the synthetic potential of 2H-azirine-2-carboxylic esters **10**. Previously, it was already known that optically active ester **10a** reacted with Grignard reagent as a nucleophile yielding NH-aziridine **1b27** [47,48] (Scheme 30). 

**Scheme 31:**
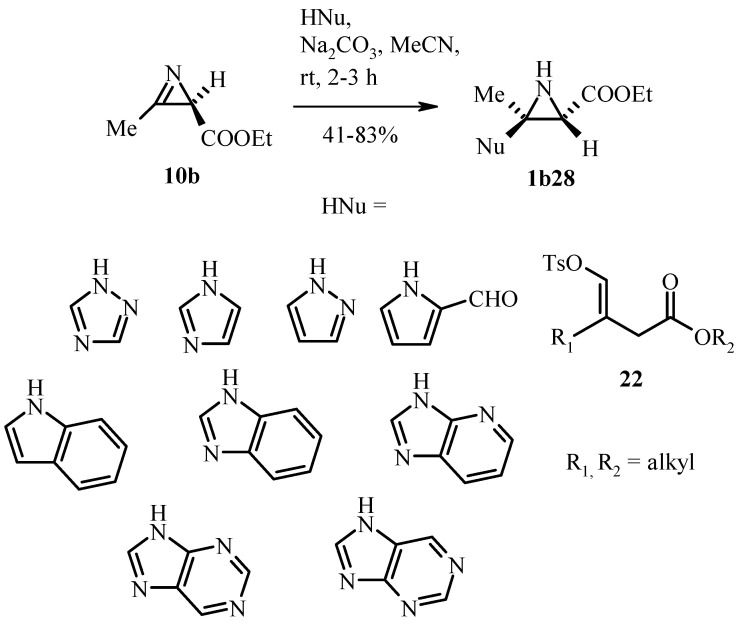
Reaction of azirines **10b** with hetarylamines.

The subsequent research by the Alves group proved that 2H-azirine-2-carboxylic ester **10b** is electrophilic enough for reaction with nitrogen nucleophiles at room temperature within some hours [49] (Scheme 31). 3-Arylated chiral aziridines **1b28** were obtained using a broad spectrum of hetarylamines as nucleophiles (nine examples). This study opened the possibility of introducing different aryl substituents for the synthesis of β-arylated aziridines. The necessary chiral azirines were produced from oximes **22** by cyclization in the presence of (+)-dihydroquinidine (Scheme 31). Chiral amine was removed from the reaction by extraction with aq. citric acid, and the obtained azirine **10b** were used in reactions with nucleophiles without further purification. 

**Scheme 32:**
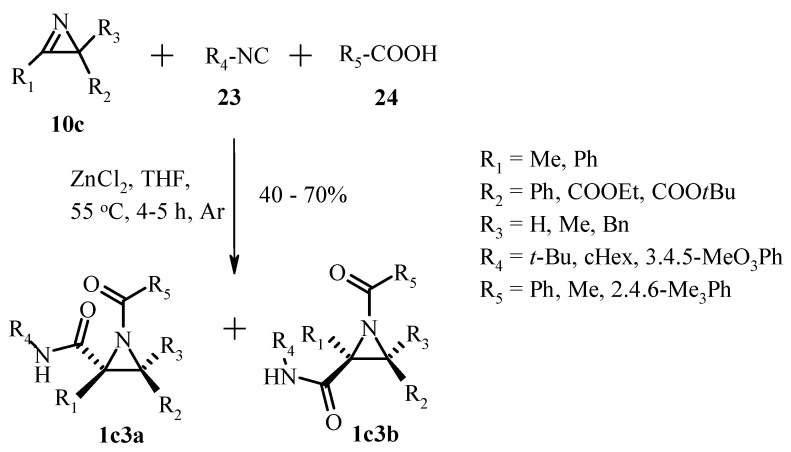
Ugi reaction of azirines **10c**.

Remarkably, 2H-azirines **10c** may serve as components in three-component Ugi reactions with izocyanide **23** and carboxylic acid **24** [50] (Scheme 32) forming aziridine-2-carboxamides **1c3**. 

This protocol allows the generation of large libraries of aziridine-2-carboxamides **1c3**, including 3-aryl, which are suitable for medical chemistry applications. The given study [50] contains a full and complete development of the practical synthetic method for products **1c3**. Firstly, the authors performed a catalyst screening and found that zinc (2) chloride was the best catalyst. Further, the reaction conditions were optimized, finding the optimal combination: THF solvent and 55 °C temperature during 4–5 h. Finally, researchers screened the reaction components (isocyanides **23**, carboxylic acids **24**, and azirines **9d**) in a large series of more than 40 examples. 3-Arylated aziridine products **1c3a**, **1c3b** were demonstrated in eight examples with moderate to good yields.

In general, the listed series of examples confirms 2H-azirines as promising substrates for aziridine construction via different approaches. 

Summarizing the information about 3-arylated aziridine synthesis from other heterocycles, we note that these methods allow complex aziridine-containing structures to be obtained, including highly functionalized products, fused ring structures, and single isomers.

## 4. Perspective Photo- and Electrochemical Methods in the Synthesis of 3-Arylated Aziridines

**Scheme 33:**
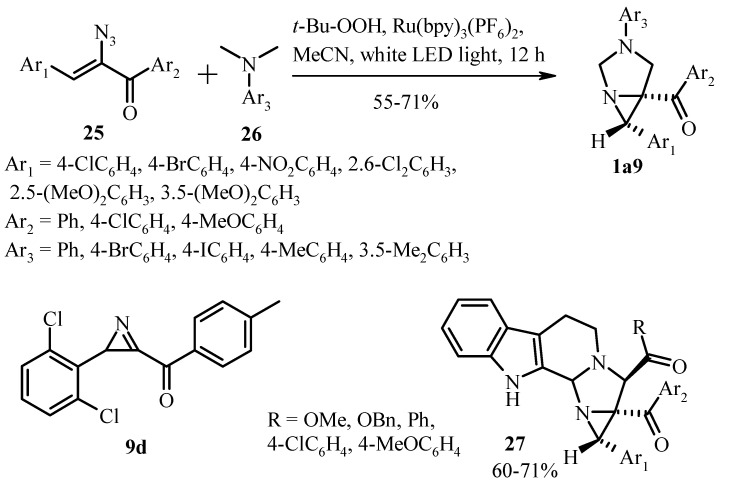
Photocascade reaction of azides **25**.

Besides the general sustainability improvements mentioned above (no chromatography for product isolation; solvent-free and mild reaction conditions), some interesting and specific physical chemistry-based approaches in the synthesis of 3-arylated aziridine products have been reported. Thus, α-keto vinyl azides **25** react in the presence of *tert*-buthyl hydroxy peroxide and photocatalyst tris-(2.2′-bipyridine)ruthenium (II) hexafluorophosphate with 1.2.3.4-tetrahydro-β-carbolines [51] or dimethylanilines **26** [52] under white LED light irradiation forming corresponding bicyclic aziridine products **1a9** [52] (20 examples) or more complex fused aziridine systems **27** [51] (18 examples) in good yields (Scheme 33). 

Studies of the reaction mechanism were performed [52]. It was proved in a single example that, in the presence of visible light and ruthenium photocatalyst, α-azidochalcone **25** got converted into 2H-azirine **9d** via photosensitized decomposition. Then 2H-azirine 9d underwent [3 + 2] cycloaddition to azomethine ylide formed in the oxidation of amine **26**. This explained the stereoselectivity of the demonstrated reaction. In the practical aspect, the authors reported the possibility of performing these reactions in a capillary flow microreactor instead of a batch. This shortened reaction time from 12 h to approx. 1 h.

**Scheme 34:**
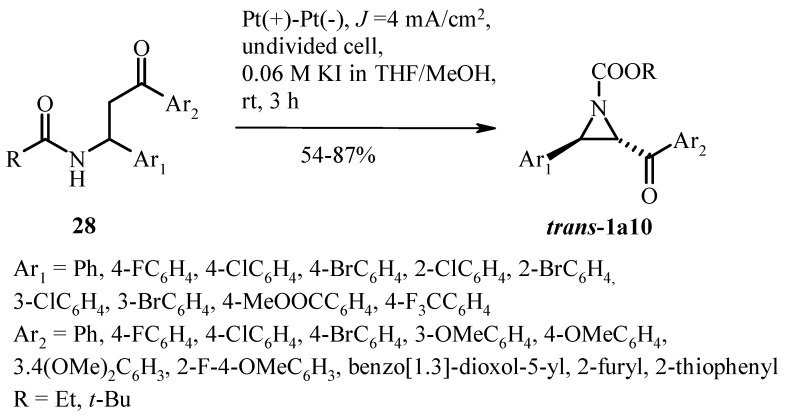
Electrochemical intramolecular amination of substrates **28**.

Excluding external oxidants in electrochemical intramolecular oxidative dehydrogenative amination of substrates **28** has been reported [53] (Scheme 34). The resulting 3-arylated aziridines ***trans*-1a10** were obtained in good yields.

Optimization of the reaction conditions and broad substrate scope (24 examples) has been demonstrated. The reaction procedure shows high sustainability because the only byproduct is hydrogen. To turn this procedure into a practical method, gram-scale synthesis of product ***trans*-1a10** (Ar1 = Ar2 = Ph, R = Et) was demonstrated in a single example (2.15 g, 73%).

In summary, physical chemistry approaches allow the construction of aziridines more cleanly and economically. Therefore these procedures seem to be promising for further investigations.

## 5. Conclusions

The current literature analysis shows that in selected cases, specific non- aziridination-based methods for obtaining 3-arylated aziridine-2-carboxylates, carboxamides, and 2-aziridinylketones are useful and hold remarkable synthetic potential. The classical cyclization was widely used for different applications in medical chemistry, e.g., to screen for potential antibacterial and antifungal active compounds in 3-arylaziridine series. The most interesting reactions are the transformations of other heterocycles into aziridines, such as the Baldwin rearrangement of isoxazolines and the use of 2H-azirines as 1.3-dipolarophiles and dienophiles in Diels-Alder cycloadditions. These methods exhibit remarkable regio- and stereoselectivity and are very simple from the practical point of view—reactions often can be performed at room temperature and without catalysts. The high functionalization possibilities are notable, yielding fused polycyclic and highly substituted aziridine derivatives if corresponding substrates are available. Another application of the high reactivity of azirines is represented by nucleophilic addition reactions and, more recently, three-component Ugi reactions with 2H-azirines as a component. This azirine chemistry is not very widely known but highly promising. Perspective research directions include both the described chemical and promising photo- and electrochemical approaches. Comparing the analyzed set of synthetic pathways with aziridination, complementarity is evident. Thus, aziridination and classical cyclization methods are more general. However, other methods mentioned in the current review show good results in specific cases for the synthesis of complex highly substituted 3-arylaziridine moiety-containing molecules.

## Data Availability

Not applicable.
